# The Greatwall-Endosulfine-PP2A/B55 pathway controls entry into quiescence by promoting translation of Elongator-tuneable transcripts

**DOI:** 10.21203/rs.3.rs-3616701/v1

**Published:** 2023-12-05

**Authors:** Javier Encinar del Dedo, Rafael López-San Segundo, Alicia Vázquez-Bolado, Jingjing Sun, Natalia García-Blanco, M. Belén Suárez, Patricia García, Pauline Tricquet, Jun-Song Chen, Peter C. Dedon, Kathleen L. Gould, Elena Hidalgo, Damien Hermand, Sergio Moreno

**Affiliations:** 1 Instituto de Biología Funcional y Genómica, CSIC, University of Salamanca, 37007 Salamanca, Spain.; 2 Antimicrobial Resistance Interdisciplinary Research Group, Singapore-MIT Alliance for Research and Technology, Singapore, Singapore.; 3 Instituto de Biología Funcional y Genómica, University of Salamanca, CSIC, 37007 Salamanca, Spain.; 4 Departamento de Microbiología y Genética, University of Salamanca, 37007 Salamanca, Spain.; 5 URPHYM-GEMO, University of Namur, rue de Bruxelles, 61, Namur 5000, Belgium.; 6 Department of Cell and Developmental Biology, Vanderbilt University School of Medicine, Nashville, United States.; 7 Department of Biological Engineering and Center for Environmental Health Science, Massachusetts Institute of Technology, Cambridge, MA, United States.; 8 Oxidative Stress and Cell Cycle Group, Universitat Pompeu Fabra, 08003 Barcelona, Spain.; 9 Lead contact.

**Keywords:** Quiescence, Nitrogen starvation, TORC1, TORC2, Greatwall, Endosulfine, PP2A/B55, tRNA modifications, Elongator, translation

## Abstract

Quiescent cells require a continuous supply of proteins to maintain protein homeostasis. In fission yeast, entry into quiescence is triggered by nitrogen stress, leading to the inactivation of TORC1 and the activation of TORC2. Here, we report that the Greatwall-Endosulfine-PPA/B55 pathway connects the downregulation of TORC1 with the upregulation of TORC2, resulting in the activation of Elongator-dependent tRNA modifications essential for sustaining the translation programme during entry into quiescence. This process promotes U_34_ and A_37_ tRNA modifications at the anticodon stem loop, enhancing translation efficiency and fidelity of mRNAs enriched for AAA versus AAG lysine codons. Notably, some of these mRNAs encode inhibitors of TORC1, activators of TORC2, tRNA modifiers, and proteins necessary for telomeric and subtelomeric functions. Therefore, we propose a novel mechanism by which cells respond to nitrogen stress at the level of translation, involving a coordinated interplay between the tRNA epitranscriptome and biased codon usage.

## Introduction

Most cells in living organisms rest in a non-dividing state called the G_0_ phase, also known as quiescence. Quiescent cells can re-enter the cell cycle with full viability when provided with the appropriate signals. Examples of quiescent cells include stem cells, neuronal progenitor cells, memory T cells, eggs, and spores. Despite their importance, the molecular mechanisms governing quiescence entry, its maintenance, and exit are not yet fully understood.

In nature, unicellular organisms continuously enter and exit quiescence depending on nutrient availability. In fission yeast, entry into quiescence is induced by nitrogen starvation, resulting in the inactivation of TOR complex 1 (TORC1) and the activation of TOR complex 2 (TORC2). TORC1 promotes cell growth in response to nutrients, growth factors, or cellular energy ^1,2^, while TORC2 is required for the nutrient stress response, cell survival during quiescence, and cell differentiation ^3–5^. The activation of the Greatwall-Endosulfine switch upon TORC1 inactivation leads to inhibition of the PP2A/B55 protein phosphatase, which is necessary for switching on TORC2 activity by increasing Gad8 phosphorylation ^4–7^.

TORC2 activation also regulates protein translation by controlling tRNA modifications through the Elongator complex ^8^. Elongator is a multiprotein complex that modifies the anticodon stem loop of tRNA^Lys^_UUU_, tRNA^Glu^_UUC_ and tRNA^Gln^_UUG_ by introducing an acetyl group at position 5 of U_34_ (cm^5^U_34_), which is further modified by Trm112-Trm9, a methyltransferase complex involved in the formation of mcm^5^U_34_, and by Ctu1-Ctu2 complex, which catalyses the thiolation at carbon 2 of U_34_ (mcm^5^s^2^U_34_). These U_34_ modifications counteract codon misreading resulting from low effective stacking interactions between A-U bases ^9–11^. They also play a crucial role in maintaining translational fidelity under stress conditions ^12–15^. Thus, Elongator is necessary for the efficient translation of mRNAs with a high AAA codon usage.

Previous studies have reported a feedback loop between the TORC1-TORC2 signalling cascade and the Elongator complex. In this loop, Elongator plays an essential role in the translation of key components of TORC2 and repressors of TORC1. Additionally, the TORC2 pathway functions as an activator of Elongator by down-regulating Gsk3, a glycogen synthase kinase that inhibits Elongator by phosphorylating the Elp4 subunit at Serine114 ^8,16,17^.

In this study, we report that elevated PP2A/B55 phosphatase activity, resulting from the deletion of Endosulfine (*igo1*Δ), impairs the translation efficiency of mRNAs enriched in AAA codons during entry into quiescence. Additionally, we demonstrate a physical and functional interaction between PP2A/B55 and Gad8, Trm112, Ctu1, and the Elongator complex. Furthermore, hyperactivation of PP2A/B55 protein phosphatase reduces the function of the Elongator complex and the amount of Trm112, Ctu1 and Cgi121 proteins, which are essential for U_34_ and A_37_ tRNA modifications at the anticodon stem loop. This reduction in translational efficiency leads to decreased protein levels from transcripts containing high AAA codon usage, such as *rap1*, *sgo2*, *clr2*, or *clr3*, all of which are crucial for telomeric and subtelomeric organisation. This induces telomeric detachment, upregulation of subtelomeric gene expression, and eventually, cell death. Our work suggests that the Greatwall-Endosulfine-PP2A/B55 pathway governs the translational programme during entry into quiescence through the control of U_34_ and A_37_ tRNA modifications. We propose that the implementation of an alternative gene expression programme in response to nitrogen starvation is based on translation of mRNAs enriched in sub-optimal AAA codons by activation of tRNA-modifying complexes.

## Results

### The Greatwall-Endosulfine switch regulates telomere silencing and telomere attachment to the nuclear envelope

**Fig. 1.**
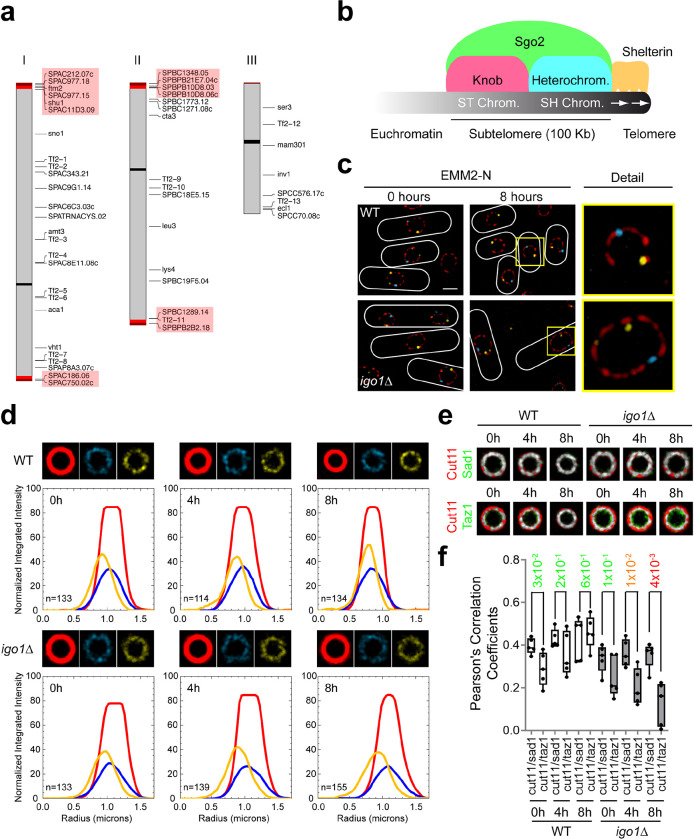
The Greatwall-Endosulfine switch regulates subtelomeric gene silencing and telomeric anchoring to the nuclear envelope. **a** Schematic representation of transcriptionally upregulated genes in the Endosulfine (*igo1Δ*) mutant. Genes overexpressed more than 10-fold in *igo1*Δ cells compared to the wild-type after 4 hours in nitrogen-free EMM2 medium. Subtelomeric genes are highlighted in red. **b** Schematic illustration of *S. pombe* subtelomeric chromatin structure (modified from ^84^). **c** Representative Super-Resolution Radial Fluctuations (SRRF) micrographs of wild-type (WT) and *igo1*Δ cells expressing Cut11:mCherry, Sad1:CFP and Taz1:YFP in nitrogen-rich EMM2 media (0 hours) and after 8 hours of nitrogen starvation in EMM2-N. The merged image and a detail view are shown. Bar: 2 μm. **d** Radial Profile Analysis for WT and *igo1*Δ cells after 0, 4 or 8 hours of nitrogen deprivation (see details in Supplementary Fig. 1b). The average projection signals for the NE (in red), the SPB (in cyan) and the telomeres (in yellow) are shown. The graphs represent the normalized integrated intensity as a function of distance in microns. The red lines correspond to the NE signal, the cyan lines correspond to the SPB signal and the yellow lines correspond to the telomeric signal. Over 100 nuclei were analysed at each time point. **e** Overlay between the average projection signals for Cut11/Sad1 or Cut11/Taz1 in WT and *igo1*Δ cells during entry into quiescence. The images were generated by projecting at least 100 nuclei. **f** Co-localization between Cut11/Sad1 and Cut11/Taz1 signals was quantified as Pearson correlation coefficients using ImageJ software. Student’s t-test p-values are indicated, significant differences are in orange or red.

To understand the function of the Greatwall-Endosulfine-PP2A/B55 pathway during entry into quiescence we compared the transcriptome of the wild-type (WT) and the Endosulfine mutant (*igo1*Δ) by RNAseq after shifting cells from nitrogen-rich (EMM2) to nitrogen-free (EMM2-N) media at times 0 and 4 hours. In nitrogen-rich medium (EMM2, time 0) the transcriptome was almost identical between the two strains. However, after 4 hours of nitrogen starvation, we found significant changes in subtelomeric gene expression, as *igo1*Δ cells showed a high expression level (more than 10-fold) of a group of subtelomeric genes in chromosomes I and II compared to WT cells (Fig. 1a; Supplementary Table 1). Similar results were obtained when we analysed the transcriptome of the Greatwall (*ppk18*Δ *cek1*Δ) mutant (Supplementary Fig. 1a; Supplementary Table 2), consistent with the fact that the Greatwall-Endosulfine-PP2A/B55 is a linear pathway ^4^. These results suggest that downregulation of PP2A/B55 plays a key role in transcriptional silencing of subtelomeric genes during quiescence entry.

The ends of chromosomes I and II are composed of telomeric repeats and the subtelomeric regions. While the telomeric repeats extend approximately 300 bp, the subtelomeric regions consist of about 100 kilobases between the telomeric repeats and the euchromatin (Fig. 1b). The heterochromatin present in the subtelomeric regions can be divided into SH chromatin, characterised by highly methylated histone H3K9, and ST chromatin, in which histone modifications are kept at low levels, but exhibit highly condensed chromosome structures called *knobs* ^18–22^. Several protein complexes essential for maintaining the telomeric and subtelomeric structure have been identified. For example, Rap1 (a component of the shelterin complex) and Bqt4 (a component of the bouquet complex) create a molecular link between telomeres and the nuclear envelope ^23–26^. Proteins such as Swi6, the SHREC complex or the CLRC complex play a role in H3K9 methylation, control nucleosome maintenance and genome stability ^27,28^. Finally, shugoshin 2, Sgo2, is an essential protein for condensation of ST chromatin and *knob* stability ^21,22^ (Fig. 1b).

To study the role of the Greatwall-Endosulfine-PP2A/B55 pathway in telomeric organisation during quiescence, we analysed nuclear-telomeric attachment in the wild-type (WT) and in the Endosulfine (*igo1Δ*) mutant in nitrogen-rich media (EMM2) and after 8 hours of nitrogen starvation (EMM2-N) using Super-Resolution Radial Fluctuations (SRRF) microscopy. Wild-type and *igo1Δ* cells tagged with Cut11-mCherry (a nuclear envelope -NE- marker), Sad1-CFP (a spindle pole body -SPB- marker) and Taz1-YFP (a telomeric marker), showed no significant differences in nitrogen-rich media. In contrast, after 8 hours of nitrogen starvation, the *igo1Δ* mutant showed telomeric detachment from the NE (Fig. 1c). To analyse the defect of the *igo1Δ* mutant in more detail, we combined SRRF microscopy with Radial Profile Analysis (see details in Supplementary Fig. 1b and Methods). The wild-type and *igo1Δ* mutant showed a perfect overlap between the NE signal (red line) and the SPB signal (blue line). However, we detected differences in the telomeric signals (yellow line) between strains. While in the wild-type strain the three signals overlapped more with time (4 and 8 hours of nitrogen starvation), in the *igo1Δ* mutant, the telomeric signal separated from the NE and the SPB signals (Fig. 1d). A similar result was obtained when we analysed the overlap between the mean NE signal and the mean SPB signal or the mean NE signal and the mean telomeric signal at different time points (Fig. 1e). Pearsońs correlation coefficients allowed us to identify significant differences between Cut11/Sad1 and Cut11/Taz1 signals in the wild-type and the *igo1Δ* mutant (Fig. 1f). These results indicate that the interaction between the NE and telomeres is lost in the *igo1Δ* mutant.

### Telomeric detachment is mediated by reduced levels of the Rap1 protein, a component of the shelterin complex

**Fig. 2.**
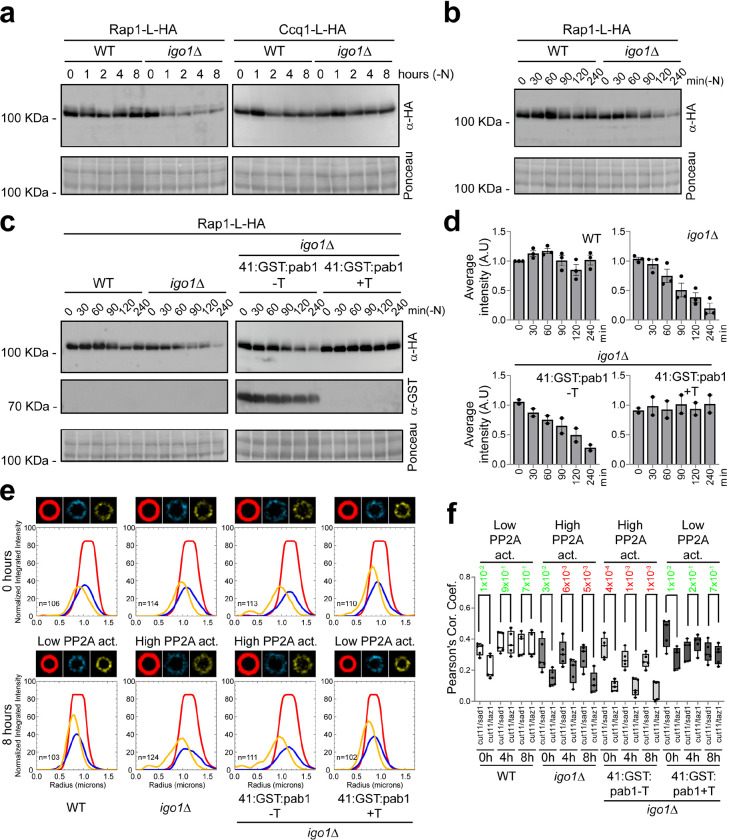
Telomeric detachment from the nuclear envelope in *igo1*Δ cells is mediated by reduced Rap1 protein levels. **a** Extracts from *rap1:L:HA* and *ccq1:L:HA* cells in a WT and *igo1*Δ background were collected at 0, 1, 2, 4 and 8 hours of nitrogen starvation. These extracts were analysed by SDS-PAGE and western blotting using anti-HA antibodies. Ponceau staining was used as the loading control. **b** Extracts from *rap1:L:HA* cells in a WT and *igo1*Δ background, collected every 30 minutes during the first 2 hours and then at 4 hours of nitrogen starvation. These extracts were analysed by SDS-PAGE and western blotting using anti-HA antibodies. Ponceau staining was used as the loading control. **c** Extracts from *rap1:L:HA* and *rap1:L:HA P*_*nmt*_*41x:GST:pab1* cells in a WT and *igo1*Δ background were collected during nitrogen starvation and analysed by SDS-PAGE and western blot using anti-HA and anti-GST antibodies. Strains were grown with or without thiamine (+T or −T) to repress or induce the *pab1* gene, encoding the B55 regulatory subunit of PP2A. Ponceau staining was used as the loading control. **d** Immunoblot quantification of **c** was performed with Image Studio Lite software from at least two independent experiments. **e** Radial Profile Analysis of WT, *igo1*Δ and *igo1*Δ *P*_*nmt*_*41x:GST:pab1* cells bearing Cut11 (in red), Sad1 (in cyan) or Taz1 (in yellow) in EMM2 (0 hours) and after 8 hours of nitrogen starvation. The *igo1*Δ *P*_*nmt*_*41x:GST:pab1* cells were grown with or without thiamine (+T or −T) to repress or induce the expression of *pab1*. Over 100 nuclei were projected to generate the images and graphics. **f** Co-localization between Cut11/Sad1 and Cut11/Taz1 signals of **e** was quantified as Pearson correlation coefficients using ImageJ software. Student’s t-test p-values are indicated, significant differences are in red.

A high level of PP2A/B55 activity, caused by deleting *igo1*, delays entry into mitosis during vegetative growth when fission yeast cells are shifted from a nitrogen-rich to a nitrogen-poor medium, or during entry into quiescence ^4^. Therefore, it seemed possible that elevated PP2A/B55 activity caused telomeric detachment during entry into quiescence. In *S. pombe*, two different complexes have been described as essential for maintaining telomeric-NE attachment, the bouquet complex and the shelterin complex. Interestingly, two subunits of the shelterin complex, Rap1 and Ccq1, have been described as heavily phosphorylated proteins ^25,26,29,30^. Thus, we considered that the phosphorylation state of these proteins might be affected by the high PP2A/B55 phosphatase activity in the *igo1Δ* mutant, triggering telomeric detachment. However, we did not detect changes in the phosphorylation state of either Rap1 or Ccq1. Surprisingly, we detected a dramatic reduction in the amount of Rap1 protein levels in the *igo1Δ* mutant, while the Ccq1 levels remained constant during the experiment (Fig. 2a). To confirm this data and improve our temporal resolution, we repeated the experiment taking samples every 30 minutes during the first 2 hours and then after 4 hours of nitrogen starvation. Once more, we detected a very significant decrease in Rap1 protein levels after 2–4 hours of nitrogen deprivation in the *igo1Δ* mutant (Fig. 2b; Fig. 2c, left panel).

Previous results from our lab have shown that *igo1Δ* phenotypes could be restored by decreasing PP2A/B55 activity ^31^, including the reduction in viability during quiescence (Supplementary Fig. 2a). This prompted us to investigate whether a reduction of PP2A/B55 activity could restore Rap1 protein levels. To modulate PP2A/B55 activity, we placed the *pab1* open reading frame, encoding the PP2A B55 regulatory subunit, under the control of the thiamine-repressible *nmt41* promoter at its chromosomal locus. We found that repressing Pab1 production and therefore PP2A/B55 activity reinstated Rap1 protein levels (Fig. 2c). Data quantification further confirmed the restoration of Rap1 protein levels when PP2A/B55 activity was reduced (Fig. 2d).

To investigate whether reducing PP2A/B55 activity could also prevent telomeric detachment *in vivo*, we examined the localization of Taz1-YFP in strains exhibiting low PP2A/B55 activity (wild-type and *igo1Δ nmt41:GST:pab1* + Thiamine) in comparison to strains with elevated PP2A/B55 activity (*igo1Δ* and *igo1Δ nmt41:GST:pab1* − Thiamine) during nitrogen starvation. Our analysis showed that low PP2A/B55 activity restored the telomeric detachment phenotype, whereas high PP2A/B55 activity maintained the telomeric attachment defect (Fig. 2e; Supplementary Fig. 2b). Statistical analysis of the data confirmed that reduced PP2A/B55 activity during nitrogen starvation was necessary for preserving telomeric organisation during quiescence (Fig. 2f). In summary, these findings suggest that the downregulation of PP2A/B55 activity during entry into quiescence is crucial for maintaining Rap1 protein levels and for anchoring telomeres to the NE.

### Sgo2, Clr2 and Clr3 protein levels are reduced in quiescent *igo1Δ* mutant cells

**Fig. 3.**
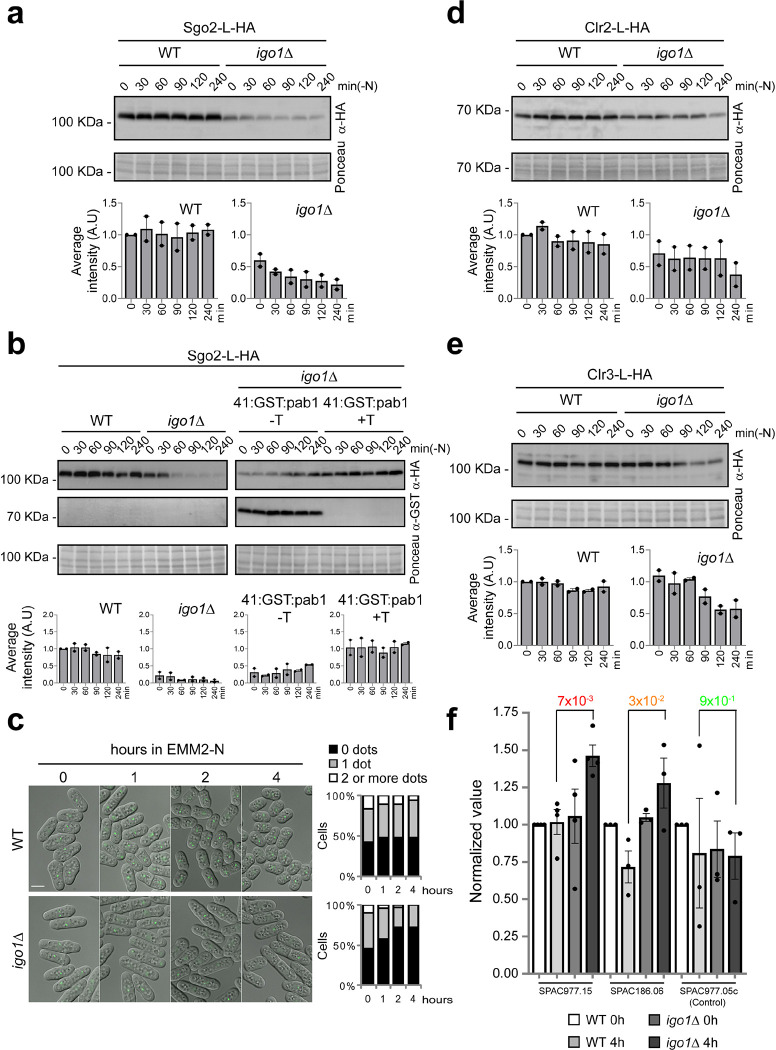
Crucial proteins required for silencing subtelomeric gene expression are downregulated in *igo1*Δ cells. **a** Extracts from *sgo2:L:HA* cells in a WT and *igo1*Δ backgrounds were collected every 30 minutes during the first 2 hours and then at 4 hours of nitrogen starvation. These extracts were analysed by SDS-PAGE and western blotting using anti-HA antibodies. Ponceau staining was used as the loading control. Immunoblot quantification was performed using Image Studio Lite software from at least two independent experiments. **b** Extracts from strains bearing *sgo2:L:HA* or *sgo2:L:HA P*_*nmt*_*41x:GST:pab1*, were analysed by SDS-PAGE and western blotting with anti-HA and anti-GST antibodies. The *igo1*Δ *P*_*nmt*_*41x:GST:pab1* cells were grown with or without thiamine (+T or −T) to repress or induce the expression of *pab1*. Ponceau staining was used as the loading control. Immunoblot quantification was performed with Image Studio Lite software from at least two independent experiments. **c** Representative micrographs of WT or *igo1*Δ cells expressing *sgo2:L:GFP* during entry into quiescence. The overlay of fluorescence and DIC images is shown. Quantification was carried out using ImageJ software from two independent experiments involving more than 150 cells. Bar: 5 μm. **d** Similar to (**a**), *clr2:L:HA* protein was analysed in both WT and *igo1*Δ backgrounds. **e** Similar to (**a**), *clr3:L:HA* protein was analysed in both WT and *igo1*Δ backgrounds. **f** ChIP-qPCR was performed with anti-H3K14-acetyl antibodies and quantified with primer pairs at the indicated ORFs. WT and *igo1*Δ cells grown in nitrogen-rich media (EMM2) or after 4 hours of nitrogen starvation were analysed. The graphs represent normalized values, and error bars (SD) for all ChIP-qPCR experiments were calculated from biological triplicates.

Different protein complexes coordinately maintain chromatin silencing in subtelomeric regions during quiescence in fission yeast. One of the critical factors for this regulation is shugoshin 2, Sgo2. Sgo2 is essential for the formation of *knobs*, highly condensed chromatin structures organised close to the ends of chromosomes I and II ^21^. Lack of Sgo2 (*sgo2*Δ) induces transcription of genes located at subtelomeric regions on chromosomes I and II ^22^, similar to what we observed in cells lacking Endosulfine (*igo1*Δ) or Greatwall (*ppk18*Δ *cek1*Δ) after nitrogen starvation (Fig. 1a; Supplementary Fig. 1a; Supplementary Tables 1, 2). This correlation prompted us to investigate whether Sgo2 levels might be altered in the *igo1*Δ mutant. Western-blot analysis revealed that *igo1*-deleted cells show a severe reduction of Sgo2 levels during entry into quiescence (Fig. 3a). As in the case of Rap1, reducing Pab1 levels in the *igo1*Δ mutant restored Sgo2 levels (Fig. 3b). To confirm the role of Igo1 in maintenance of Sgo2 levels and *knob* formation, we studied the localisation of Sgo2 during quiescence entry. Sgo2 protein was tagged with GFP and its localisation during nitrogen starvation was examined. As previously described, in the wild-type strain, Sgo2-GFP localised as nuclear dots in most cells, ranging from 1 to 3 dots per cell (Fig. 3c). In the *igo1*Δ mutant, we detected no significant differences with the wild-type in nitrogen-rich media (t=0 hours), only a slight decrease in dot size and brightness. However, when we shifted the cells to nitrogen-free media we observed a clear decrease in the number of dots per cell in the *igo1*Δ mutant (Fig. 3c). These data indicate that Igo1 is required for maintaining Sgo2 protein levels and for the formation of *knobs*, a structure essential for maintaining the transcriptional repression of subtelomeric genes.

Another key protein complex required for silencing subtelomeric regions is the heterochromatic repressor complex SHREC (Snf2-like/HDAC-containing repressor complex) ^32,33^, composed of Mit1, Clr1, Clr2, and Clr3. The SHREC complex plays regulatory roles in histone acetylation, as a chromatin remodeller and in the stability of subtelomeric nucleosomes ^27,32,33^. To determine if SHREC was also affected by PP2A activity, we examined Clr2 and Clr3 levels. In both cases, we detected a modest but reproducible decrease in protein levels during nitrogen starvation in the *igo1Δ* mutant (Fig. 3d,e). ChIP analysis showed that lack of Igo1 caused an increase in histone H3-K14 acetylation in the overexpressed subtelomeric genes SPCC977.15 and SPAC186.06 after 4 hours of nitrogen starvation consistent with loss of SHREC function (Fig. 3f). These results suggest that the Greatwall-Endosulfine-PP2A/B55 pathway modulates SH chromatin organisation and subtelomeric gene silencing.

### PP2A/B55 regulates translation through its physical and functional interaction with protein complexes involved in tRNA modification

Our data indicate that the *igo1Δ* mutant exhibits reduced levels of proteins essential for maintaining telomeric and subtelomeric organisation. However, what is the molecular mechanism underlying these phenotypes? We explored three possibilities: reduced protein stability, reduced transcription, or reduced translation.

To examine protein stability, we treated cells with cycloheximide and monitored Rap1 protein levels over time. After treatment with cycloheximide, Rap1 was degraded with similar kinetics in wild-type and *igo1Δ* cells (Supplementary Fig. 3a). Similarly, *rap1* mRNA levels were not reduced in *igo1Δ* cells; on the contrary, the *rap1* gene exhibited higher transcript levels in the *igo1Δ* mutant compared to wild-type cells (Supplementary Fig. 3b).

Evidence of a translation defect in the *igo1*Δ mutant was obtained by mass-spectrometry analysis of proteins co-purifying with the PP2A/Pab1 protein phosphatase. Paa1, the structural subunit of the PP2A complex, was tagged with YFP and expressed from its endogenous promoter at its chromosomal locus. After one hour of nitrogen starvation, Paa1-YFP was pulled down and co-purifying proteins were analysed by mass-spectrometry. Our results revealed the presence of all components of PP2A protein complexes including Paa1, the catalytic subunits Ppa1, Ppa2, and Ppa3, and the regulatory subunits Pab1, Par1 and Par2 (Supplementary Table 3). Additionally, several PP2A regulators (Igo1, Zds1, Dis2, Ppe1 and Ekc1) and components of the PP2A SIP/STRIPAK complex ^34^ were also detected, confirming that the pull-down approach was successful.

**Fig. 4.**
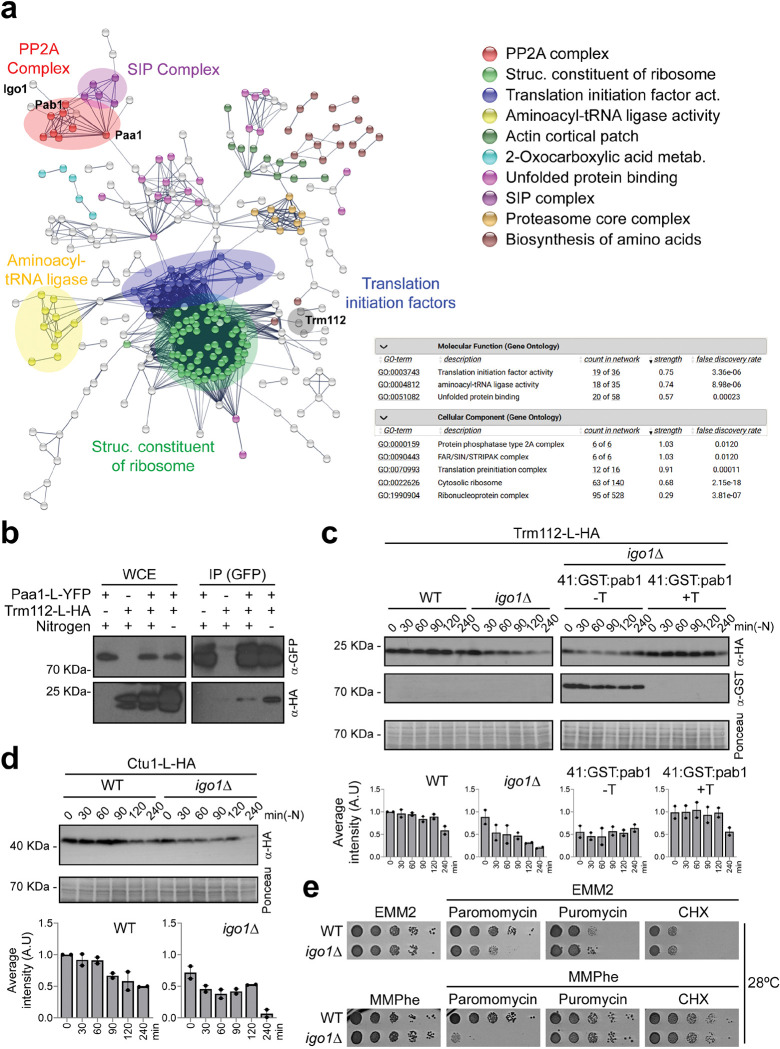
PP2A interacts with proteins involved in tRNA modification. **a** Interacting network resulting from mass-spectrometry analysis for *paa1:L:YFP* in EMM-N. Cellular processes or protein complexes with a significant enrichment are colour-coded. **b** Interaction between *paa1:L:YFP* and *trm112:L:HA*. Protein extracts from cells expressing *paa1:L:YFP*, *trm112:L:HA* or *paa1:L:YFP trm112:L:HA* were immunoprecipitated in nitrogen-rich or nitrogen-depleted media with anti-GFP beads and probed with anti-GFP and anti-HA antibodies. Extracts (WCE) were assayed for levels of *paa1:L:YFP* and *trm112:L:HA* by western blot. **c** Extracts from cells expressing *trm112:L:HA* or *trm112:L:HA P*_*nmt*_*41x:GST:pab1*, were analysed by SDS-PAGE followed by immunoblotting with anti-HA and anti-GST antibodies. The *igo1*Δ *P*_*nmt*_*41x:GST:pab1* cells were grown in EMM2 with or without thiamine (+T or −T) to repress or induce the expression of *pab1*. Ponceau staining was used as the loading control. Immunoblot quantification was performed with Image Studio Lite software from at least two independent experiments. **d** Extracts from *Ctu1:L:HA* cells in a WT and *igo1*Δ backgrounds were collected every 30 minutes during the first 2 hours and then at 4 hours of nitrogen starvation. These extracts were analysed by SDS-PAGE and immunoblotting using anti-HA antibodies. Ponceau staining was used as the loading control. Immunoblot quantification was performed using Image Studio Lite software from at least two independent experiments. **e** Serial dilutions from WT and *igo1*Δ cultures were spotted onto EMM2 (Minimal Media containing NH_4_Cl) or MMPhe (Minimal Media containing Phenylalanine) plates without or with paromomycin (0.5 mg/ml), puromycin (0.5 mg/ml) or cycloheximide (CHX, 2.5 μg/ml).

The mass-spectrometry analysis also showed an over-representation of proteins related to ribosome structure, translation initiation, aminoacylation, and tRNA modification in the Paa1 interactome (Fig. 4a). We focused on Trm112, a widely conserved protein with a crucial role in translation. Specifically, Trm112 regulates methyltransferase enzymes (Trm9, Trm11, Mtq2 and Bud23) during ribosome biogenesis, tRNA modification and stop codon recognition ^35,36^. We confirmed an interaction between Trm112 and the PP2A-Pab1 complex by repeating the mass-spectrometry analysis using Pab1, the B55 regulatory subunit of the PP2A complex, as bait. Trm112 was pulled down as an interacting partner of PP2A/Pab1 (Supplementary Fig. 4a,b; Supplementary Table 4). The Trm112-Paa1 interaction was further validated by co-immunoprecipitation, showing a stronger association in nitrogen-depleted than in nitrogen-rich media (Fig. 4b). Interestingly, several subunits of the Elongator complex (Elp1, Elp2 and Elp3) were also pulled down as interacting partners of PP2A/Pab1 when Pab1 was slightly overexpressed from the *nmt41* promoter (Supplementary Fig. 4b,c; Supplementary Table 5).

The Elongator complex, along with Trm112/Trm9 and the Ctu1/Ctu2 complexes, plays a critical role in the formation of the 5-methoxycarbonylmethyl (mcm^5^) and 5-methoxycarbonylmethyl-2-thiouridine (mcm^5^s^2^) side chains on uridine 34 (U_34_) at the tRNA wobble position during vegetative growth and under stress conditions ^8,12,13,37–40^. We conducted an analysis of Trm112 protein levels during nitrogen starvation in both wild-type and *igo1* deleted cells. In the wild-type, Trm112 levels remained constant during the first two hours and then exhibited a slight decrease after four hours. In contrast, in the *igo1Δ* mutant, we observed a more pronounced reduction in Trm112 proteins levels during entry into quiescence (Fig. 4c, left panel). Interestingly, as shown previously for other proteins, the reduction of PP2A/B55 activity restored Trm112 protein levels (Fig. 4c, right panel). We also found that the levels of Ctu1 protein, which cooperates with Ctu2 in tRNA U_34_ thiolation ^41,42^, were also diminished in the *igo1Δ* background (Fig. 4d).

Collectively, these data suggest potential impairment of tRNA modifications in the *igo1Δ* mutant. To assess this possibility, we tested the sensitivity of the *igo1Δ* mutant to drugs that affect translation, such as paromomycin, puromycin or cycloheximide, in nitrogen-rich (EMM2) and nitrogen-poor (MMPhe) media. Among all the drugs tested, only paromomycin, which induces codon misreading ^43^, exhibited an effect on the *igo1Δ* mutant (Fig. 4e), particularly in MMPhe.

In summary, our data indicate a potential role for the Greatwall-Endosulfine-PP2A/B55 pathway in translation during the onset of quiescence. This role likely involves tRNA modifications that enhance codon-anticodon recognition.

### The *igo1Δ* mutant is defective in U_34_ and A_37_ tRNA modifications

In all organisms, modifications of uridine 34 at the wobble position (U_34_) of certain tRNAs are necessary to enhance codon-anticodon recognition ^37^. These modifications are mediated by the Elongator complex, which introduces an acetyl group at position 5 of U_34_ (cm^5^U_34_), the Trm112/Trm9 methyltransferase complex involved in the formation of mcm^5^U_34_, and the Ctu1-Ctu2 complex, which catalyses the thiolation at carbon 2 of U_34_ (mcm^5^s^2^U_34_) (Supplementary Fig. 5a).

Previous studies in *S. pombe* have reported that differences in codon usage and tRNA modifications play a crucial role in regulating translation efficiency during the cell cycle and under oxidative stress. The mRNAs of the cell cycle regulator Cdr2 ^39^ and of the stress-responsive transcription factors Atf1 and Pcr1 ^12,13^ exhibit a high usage of lysine AAA codons compared to AAG codons, and their translational rate is particularity sensitive to deficiencies in tRNA modifications mediated by the Elongator, Trm112/Trm9 and Ctu1/Ctu2 complexes ^8,12,13,39^. Therefore, we hypothesised that differences in AAA_lys_ codon usage might be responsible for the translation phenotype observed in the *igo1*Δ mutant. To test this hypothesis, we examined the use of AAA_lys_ versus AAG_lys_ codons for some of the proteins analysed in our study and found that all the proteins defective in the *igo1Δ* mutant (Rap1, Sgo2, Clr3, Clr2, and Ctu1) primarily utilise the AAA_lys_ codon (Supplementary Fig. 5b). Proteins with reduced AAA_lys_ codon usage, such as Ccq1, Pgk1, Krs1, or Swi6, did not exhibit translation deficiencies during nitrogen starvation in the *igo1*Δ mutant (Fig. 2a; Supplementary Fig. 5b,c).

**Fig. 5.**
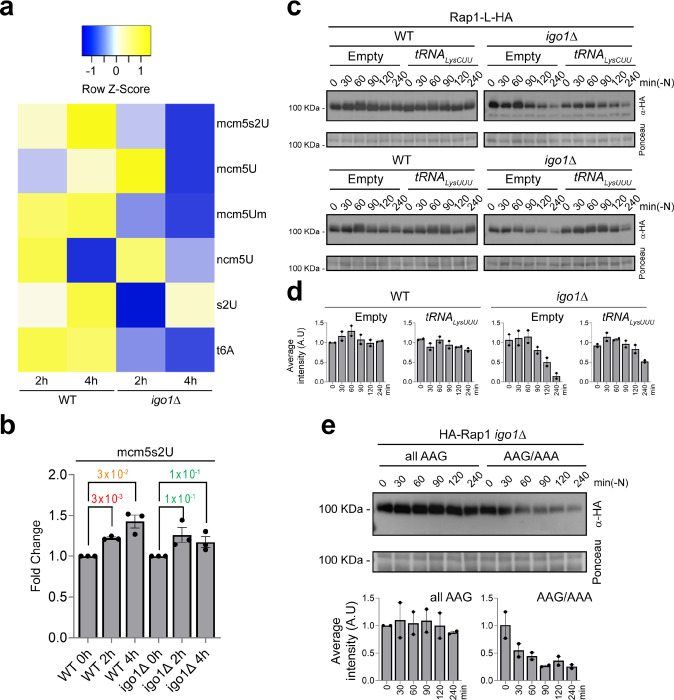
Igo1 regulates some tRNA modifications. **a** Heatmap analysis of changes in the relative levels of tRNA ribonucleoside modifications in the WT and the *igo1*Δ mutant. The side colour bar displays the range of z-score change values. The z-score was calculated as the value for each time point minus the average value for the modification, and the resulting value was divided by the standard deviation. **b** Fold-change of mcm^5^S^2^U_34_ modification in WT and *igo1*Δ mutant cells. Student’s t-test p-values were calculated from biological triplicates. **c** Extracts from strains bearing *rap1:L:HA* protein transformed with episomal plasmids ptRNA_CUULys_, ptRNA_UUULys_ or the empty vector pREP42x were analysed by SDS-PAGE and western blotting with anti-HA antibodies during nitrogen starvation. Ponceau stain was used as a loading control. **d** Immunoblot quantification performed with Image Studio Lite software from at least three independent experiments. **e** Extracts from *igo1*Δ mutant transformed with episomal plasmids *P*_*nmt*_*41x:HA:rap1* (AAA/AAG) or mutated version *P*_*nmt*_*41x:HA:rap1* (all AAG) were analysed by SDS-PAGE followed by immunoblotting with anti-HA antibodies during nitrogen starvation. Ponceau staining was used as a loading control. Immunoblot quantification were performed with Image Studio Lite software from at least two independent experiments.

To confirm the potential defect in U_34_ tRNA modification in the *igo1Δ* mutant, we employed quantitative liquid chromatography coupled to mass spectrometry (LC-MS) ^8^ to analyse tRNAs extracted from wild-type and *igo1*Δ cells. Samples were collected during exponential growth and at 2 and 4 hours of nitrogen starvation. This analysis revealed an increase in mcm^5^s^2^U_34_ levels in wild-type cells upon entry into quiescence. However, in the *igo1Δ* mutant, the levels of mcm^5^s^2^U_34_ diminished after 4 hours of nitrogen starvation (Fig. 5a,b). Furthermore, this analysis also indicated a reduction in A_37_ N6-threonylcarbamoyladenosine (t^6^) modification in the *igo1*Δ mutant (Fig. 5a; Supplementary Fig. 6a). The t^6^A_37_ tRNA modification, present in Archaea and Eukarya, mediated by the protein Sua5 and the KEOPS/EKC complex, is essential for cell growth and accurate translation ^44–46^. Once again, we hypothesised that differences in AAA_lys_ codon usage might be responsible for the reduction in t6 A_37_ modification in the *igo1*Δ mutant. When we examined the use of AAA_lys_ versus AAG_lys_ codons for Sua5 and the KEOPS/EKC subunits, we found that Pcc1 and Cgi121 (two components of KEOPS/EKC complex) primarily use the AAA_lys_ codons (Supplementary Fig. 6b). Taking Cgi121 as an example, we detected a significant reduction in Cgi121 level in the *igo1* mutant during nitrogen starvation (Supplementary Fig. 6c). These data suggest that a defect in the translation of Cgi121 protein could be responsible of the reduction in t^6^A_37_ tRNA modification in the *igo1*Δ mutant. Both mcm^5^s^2^U_34_ and t^6^A_37_ modifications are involved in decoding codons that start with adenosine, promoting codon-anticodon pairing and enhancing translation fidelity ^47,48^. These findings provide a molecular explanation for the paromomycin hypersensitivity and translation defect observed in the *igo1*Δ mutant under nitrogen-stress conditions (Fig. 4e).

As cells enter quiescence, a notable reduction in tRNA^Lys^_UUU_ levels was observed in both wild-type and *igo1*Δ cells (Supplementary Fig. 5d), suggesting that this tRNA becomes limiting in quiescent cells. Previous studies in yeast and worms have shown that over-expression of tRNAs can effectively restore translation rates and protein homeostasis in mutants defective in tRNA modification ^8,12,13,39,47^. Using Rap1 as an example, we assessed whether over-expression of tRNA^Lys^_UUU_ would lead to recovery of Rap1 protein levels in the *igo1*Δ mutant, with tRNA^Lys^_CUU_ over-expression serving as a control. As anticipated, tRNA^Lys^_CUU_ overexpression had no impact on Rap1 levels, while overexpression of tRNA^Lys^_UUU_ partially restored Rap1 protein levels (Fig. 5c, d). To confirm that the reduced levels of Rap1 protein in *igo1*Δ cells resulted from defective translation of its mRNA, we engineered a mutant version of the *rap1* gene in which the 40 AAA codons were substituted with AAG, making all lysine codons independent of tRNA modification. Consistent with previous findings for other proteins, the translation deficiency of Rap1 in the *igo1*Δ mutant was completely rescued by expressing the *rap1-allAAG* allele (Fig. 5e).

In summary, considering all the data, we conclude that during the transition into quiescence, the Endosulfine Igo1 is necessary for facilitating U_34_ and A_37_ tRNA modifications, which are critical for enhancing the translation efficiency and fidelity of proteins encoded by mRNAs with high AAA_lys_ codon usage.

### Gad8 phosphorylation is required to enhance translation during quiescence entry

We then investigated the underlying molecular mechanism that triggers the translational defect due to a failure in tRNA modification. We identified Gad8 as an interactor of PP2A/Pab1 (Supplementary Fig. 4c). Previous studies demonstrated that PP2A/Pab1 counteracts the phosphorylation of Gad8 by TORC2 at serine 546, suggesting that Gad8 is a direct target of PP2A/Pab1 ^6,7^. Additionally, active Gad8 phosphorylated at S546 activates Elongator by inhibiting Gsk3, a glycogen synthase kinase that inhibits Elongator by phosphorylating the Elp4 subunit at serine 114 ^8^.

**Fig. 6.**
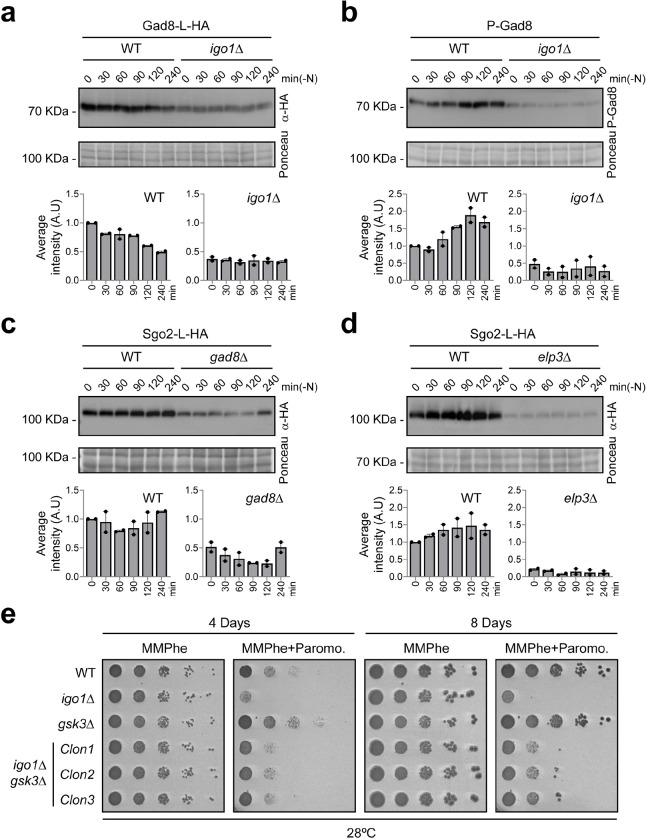
Endosulfine, Gad8 and Elongator are required for efficient translation of certain mRNAs during quiescence entry. **a** Extracts from WT and *igo1*Δ cells expressing *gad8:L:HA*, were analysed by SDS-PAGE and immunoblotting with anti-HA antibodies during nitrogen starvation. Ponceau stain was used as a loading control. Immunoblot quantification were performed with Image Studio Lite software from at least two independent experiments. **b** Same as in (**a**), Gad8 phosphorylation state was analysed in WT and *igo1*Δ cell extracts. **c** Same as in (**a**), *sgo2:L:HA* protein was analysed in a WT and *gad8*Δ cell extracts. **d** Same as in (**a**), *sgo2:L:HA* protein was analysed in a WT and *elp3*Δ cell extracts. **e** Serial dilutions from cultures of WT, *igo1*Δ, *gsk3*Δ and *igo1*Δ *gsk3*Δ were spotted onto MMPhe (Minimal Media with Phenylalanine) plates without or with paromomycin (0.5 mg/ml).

**Fig. 7.**
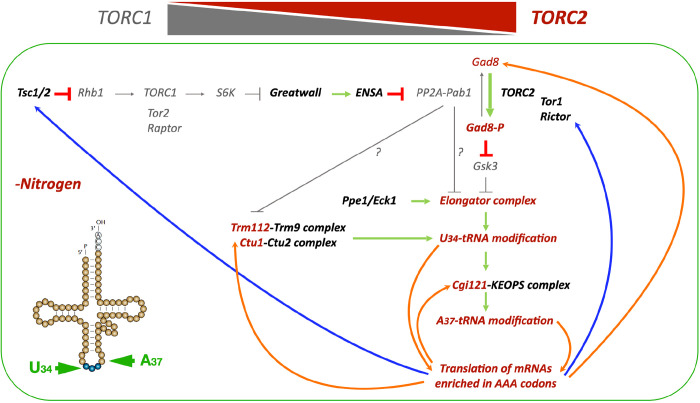
Activation of TORC2-Gad8 signalling in quiescent cells promotes the translation of mRNAs with a high AAA_Lys_ codon usage. This model is based on previous work in fission yeast, demonstrating that nitrogen starvation induces the inactivation of TORC1 and the activation of TORC2 signalling through the Greatwall-Endosulfine-PP2A/B55 pathway ^4–7^. Phosphorylation of Gad8 at S546 leads to the inhibition of Gsk3 and the activation of Elongator, which promotes U_34_ tRNA modification and translation of Tsc1, an inhibitor of TORC1, as well as activators of TORC2, such as Tor1 and Rictor (depicted by blue arrows) ^8^. In this study, we present additional feedback loops (indicated by orange arrows) that enhance the translation of Gad8, Trm112, Ctu1 and Cgi121, further increasing the U_34_ and A_37_ tRNA modifications necessary for the efficient translation of mRNAs enriched in AAA codons. Such mRNAs include *rap1*, *clr2*, *clr3* and *sgo2*, which encode proteins required for the correct attachment of telomeres to the NE.

These findings prompted us to investigate the potential role of Igo1 in Gad8 phosphorylation during quiescence entry. Notably, it has previously been reported that deletion of *elp3*, which encodes the tRNA acetyltransferase subunit of the Elongator complex, leads to a reduction in Gad8 protein levels ^8^. A sequence analysis of the *gad8* mRNA revealed a high usage of AAA_lys_ codons, like *rap1* (Supplementary Fig. 5b, *gad8* z-score_AAA/AAG_ = 0.73/−0.73 vs. *rap1* z-score_AAA/AAG_ = 0.72/−0.71). This observation led us to investigate whether high PP2A/B55 activity, in the absence of Igo1, could be relevant for maintaining Gad8 protein levels during nitrogen starvation. Western blot analysis clearly showed a decrease in Gad8 protein levels in the *igo1*Δ mutant (Fig. 6a). Furthermore, the phosphorylation status of Gad8 at S546 was also reduced in the absence of Igo1, compared to wild-type cells (Fig. 6b). These results suggest that during nitrogen starvation, inhibition of PP2A/Pab1 leads to the accumulation of phosphorylated Gad8 at S546 by TORC2 and consequent activation of the Elongator complex. This mechanism generates a positive feedback loop that enhances translation of Gad8 and promotes more U_34_ tRNA modifications (see Fig. 7).

If our model is correct, and PP2A/B55 indeed regulates Elongator activity through Gad8 protein homeostasis, the deletion of the *gad8* gene should lead to a substantial decrease in proteins with high AAA_lys_ codon usage. To test this hypothesis, we examined the levels of Sgo2, a protein with a very high AAA_lys_ codon usage (z-score_AAA/AAG_ = 1.28/−1.28), in a *gad8Δ* mutant background. Western blot analysis showed a reduction in Sgo2 protein levels in the *gad8Δ* mutant (Fig. 6c). As a positive control, we used the *elp3Δ* mutant, where the reduction in Sgo2 levels was even greater (Fig. 6d). Moreover, both *gad8Δ* and *elp3Δ* mutants displayed sensitivity to paromomycin in both nitrogen-rich and nitrogen-poor media (Supplementary Fig. 6d), although the sensitivity to paromomycin in nitrogen-poor medium was more pronounced in the *igo1*Δ mutant (Figs. 6e; Supplementary Fig. 6d). Thus, our data strongly suggests that defects in the Elongator activation pathway led to decreased translation efficiency of mRNAs with high AAA_lys_ codon usage.

Finally, to confirm the connection between Igo1 and the Elongator activation pathway, we generated a double mutant, *igo1Δ gsk3Δ*. Gsk3 is an Elongator inhibitor in *S. pombe* ^8^. As mentioned earlier, the *igo1Δ* mutant exhibited sensitivity to paromomycin, particularly in nitrogen-poor media containing phenylalanine (Fig. 4e). If this phenotype is indeed related to reduced Elongator activity, the *igo1Δ gsk3Δ* double deletion should rescue this defect. Wild-type, *igo1Δ* and *gsk3Δ* single mutants, along with the *igo1Δ gsk3Δ* double mutant, were cultivated in nitrogen-poor (MMPhe) medium with or without paromomycin, and their growth phenotype was assessed. The *igo1Δ* mutant displayed hypersensitivity to paromomycin, while the *gsk3Δ* mutant exhibited mild resistance compared to the wild-type strain. A partial improvement in cell growth in the presence of paromomycin was detected in the *igo1Δ gsk3Δ* double mutant clones compared to the *igo1Δ* mutant (Fig. 6e). These findings strongly suggest that proper Igo1-mediated activation of Elongator is crucial for maintaining the rate of translation during quiescence entry.

## Discussion

Entry into quiescence in yeasts is regulated by diverse signalling cascades that converge at the Greatwall-Endosulfine-PP2A/B55 pathway ^3,5,49–51^. In *S. pombe*, the primary signal regulating entry into quiescence is nitrogen starvation, which reduces the activity of TORC1 and increases the activity of TORC2 ^4,6,7,52–58^. Inactivation of TORC1 results in reduced protein synthesis and the activation of protein degradation through autophagy. However, quiescent cells must maintain a continuous supply of specific proteins to remain viable.

In this study, we demonstrate that the Greatwall-Endosulfine-PP2A/B55 pathway links the inactivation of TORC1 with the activation of TORC2 signalling to promote the activation of the Elongator complex and other tRNA modification complexes essential for sustaining the translation programme during quiescence. This is achieved by facilitating U_34_ and A_37_ tRNA modifications, which increase translation efficiency and fidelity of critical proteins, including those necessary for telomeric and subtelomeric functions.

The reduction of PP2A/B55 activity, achieved through the activation of Greatwall and Endosulfine, is required to accumulate phosphorylated Gad8 at S546 ^7^. This phosphorylation event increases the activity of the Elongator complex by inhibiting glycogen synthase kinase, Gsk3 ^8^. The increased Elongator activity promotes the efficient translation of mRNAs containing high AAA_lys_ codon usage, such as *tsc2* (an inhibitor of TORC1) ^8^, *gad8* (a positive effector of TORC2), *trm112*, *ctu1* and *cgi121* (involved in U_34_ and A_37_ tRNA modifications). All of these facilitate the switch from high TORC1 to high TORC2 activity as cells enter quiescence. Furthermore, the synthesis of key proteins with roles in telomeric and subtelomeric organisation, such as Rap1, Sgo2, Clr2 or Clr3, which also exhibit a high AAA_lys_ codon usage, is dependent on the correct activation of Elongator (Fig. 7).

Previous studies have demonstrated that the deletion of *rap1* (encoding a component of the shelterin complex) or *bqt4* (encoding a component of the bouquet complex) causes telomeric detachment from the NE ^23,25,26^. Our findings suggest that the telomeric detachment defect in the *igo1Δ* mutant is probably caused by a reduction in Rap1 protein levels (Fig. 2a–d). Interestingly, Bqt4, the other protein that creates a molecular link between telomeres and the NE, has a low AAA_lys_ codon usage (z-score_AAA/AAG_ = −0.94/0.95), making it unlikely to be responsible for the telomeric detachment phenotype in the *igo1Δ* mutant. However, we cannot rule out the possibility that other proteins may be involved in the telomeric attachment to the NE, such as Lem2, a member of the Lap2/Emerin/Man1 (LEM) family of lamin-associated proteins, which is known to be involved in telomere anchoring and heterochromatic gene silencing ^59–61^. Lem2 mRNA has a high AAA_lys_ codon usage (z-score_AAA/AAG_ = 0.88/−0.88), suggesting that it may also be subject to translation defects in the *igo1Δ* mutant, which could potentially affect telomere attachment to the NE.

In addition to the telomeric anchoring defect, the *igo1Δ* mutant exhibited upregulation of genes located in the subtelomeric regions of chromosomes I and II (Fig. 1a; Supplementary Fig. 1a). Several proteins related to subtelomeric organisation were defective in the *igo1Δ* background, particularly Sgo2, whose protein levels were significantly reduced (Fig. 3a). Sgo2 plays a role in *knobs* assembly, and its deletion leads to derepression of genes located in subtelomeric regions ^21,22^. Our data demonstrate that Igo1 is involved in *knobs* assembly, and its deletion results in defects in the regulation of subtelomeric genes. The overlapping roles and phenotypes between Sgo2 and Igo1 suggest that the reduction in Sgo2 protein levels is responsible for the derepression of subtelomeric genes in the *igo1Δ* mutant. However, other proteins, such as Clr2 or Clr3, which are involved in silencing could also contribute to this phenotype.

All the proteins tested in our study had their levels restored after reducing PP2A/B55 activity, indicating that low PP2A/B55 activity was necessary to maintain protein homeostasis during quiescence entry. But what is the link between PP2A/B55 activity, translation and the telomeric/subtelomeric organisation?

Previous research has shown that deleting specific components of TORC2 signalling, such as *tor1* or *gad8*, leads to a significant derepression of genes located in subtelomeric regions ^62^. However, the molecular mechanism linking these processes remained unclear. Our data reveal that not only components of the TORC2 complex but also elements acting downstream of the TORC1 complex, such as Greatwall (Ppk18 and Cek1) or Endosulfine (Igo1), play a crucial role in regulating subtelomeric gene expression. These findings establish a connection between the Greatwall-Endosulfine-PP2A/B55 pathway and telomeric/subtelomeric organisation.

A link between the TORC2 signalling pathway, the Elongator complex, and tRNA modifications has been demonstrated ^8^. Our data further demonstrate that this connection is particularly relevant during entry into quiescence by elucidating the molecular details linking nitrogen starvation, TORC1 inactivation, activation of Greatwall-Endosulfine, inactivation of PP2A/Pab1, activation of Gad8, and upregulation of tRNA-modifying complexes (Fig. 7). Indeed, these molecular events affect not only the Elongator complex but also other tRNA modifiers like Trm112 or Ctu1. These interactions create positive feedback loops that are responsible for the transition from high TORC1 to high TORC2 activity, subsequently leading to further tRNA modifications. Mutations in Greatwall (Ppk18 and Cek1) or Endosulfine (igo1) result in a defect in this transition, causing a failure in tRNA modifications that affect the translation of mRNAs with a high AAA_lys_ codon usage encoding for proteins such as Rap1, Sgo2, Clr2 and Clr3. This, in turn, triggers telomeric detachment and the derepression of subtelomeric genes.

In addition to the mcm^5^s^2^U_34_ defect, we also observed a reduction in t^6^A_37_ in the Endosulfine (*igo1*Δ) mutant (Fig. 5a). These two closely located tRNA modifications play a crucial role in ensuring accurate codon-anticodon interactions by stabilising codon-anticodon pairings ^63–66^. Interestingly, tRNA^Lys^_UUU_ carries both the mcm^5^s^2^U_34_ and t^6^A_37_ modifications, both of which are defective in the Endosulfine (*igo1*Δ) mutant. This finding provides an explanation for the strong sensitivity of *igo1*Δ cells to paromomycin compared to mutants in Elongator (*elp3*Δ) or Gad8 (*gad8*Δ). The defect in the t^6^A_37_ modification could be explained by a reduction in Cgi121 protein levels (Supplementary Fig. 6c), one of the subunits of the KEOPS complex responsible for this modification ^48,64^, which is encoded by an mRNA with a high AAA_lys_ codon usage.

Finally, an interesting concept has emerged over the last few years on tRNA modifications and stress, known as tRNA modification tuneable transcripts (MoTTs). These transcripts are characterised by a specific use of degenerate codons and codon biases to encode essential stress response proteins. Translation of these transcripts is affected by modifications at the wobble position of the tRNAs ^14,15^. Our work supports the idea that mRNAs encoding proteins involved in nutrient starvation, stress response or even the translation of viral RNA genomes present MoTTs, which allow for an increase in translation efficiency under stress conditions ^8,12,13,67–70^. One of these transcripts, the Hif1α mRNA, is translated in drug-resistant melanomas through a mechanism involving the activation of Elp1 by the PI3 kinase signalling pathway. The activation of Hif1α promotes drug resistance by inducing anaerobic glycolysis ^71^. Therefore, the mcm^5^s^2^U_34_ modification in the tRNA anticodon promotes the translation of mRNAs enriched in AAA codons, including Hif1α mRNA. This discovery opens new avenues for identifying inhibitors of Elongator and other tRNA modifiers to treat drug-resistant tumours and combat viral infections.

## Methods

### Strains and Growth Conditions

Yeast strains are listed in Supplementary Table 6. Fission yeast cells were cultured and genetically manipulated according to standard protocols ^72^. Genetic crosses were performed on malt extract agar plates. Cells were typically cultured overnight at the appropriate temperatures in yeast extract supplemented with adenine, leucine, histidine, lysine, and uracil (YES), or in Edinburgh minimal medium containing 93.5 mM ammonium chloride (EMM2) as a nitrogen source. For nitrogen starvation experiments, exponentially growing cells were shifted from Edinburgh minimal medium (EMM2) at 28°C to minimal medium without nitrogen (EMM2-N) at 25°C.

In overexpression experiments using the *nmt1*^*+*^ promoter, cells were grown to the logarithmic phase in EMM2 containing 15 μM thiamine. Then, the cells were harvested and inoculated in fresh EMM2 medium without thiamine.

### DNA Techniques and Plasmid Construction

DNA manipulations were performed as described in Sambrook, J., Fritsch, E.F., and Maniatis, T. (1989). Enzymes for molecular biology were obtained from Fermentas and Thermo Fisher. PCRs were performed with using Velocity DNA polymerase (Bioline). Oligonucleotides employed for strain and plasmid construction are listed in Supplementary Table 7. Information regarding construction strategies is available upon request. Plasmids used in this study carry the ampicillin resistance gene for selection in *E. coli* and are listed in Supplementary Table 7.

### RNA Isolation, RNAseq and RT-qPCR

Wild type (2666), *igo1*Δ (2727) and *ppk18*Δ *cek1*Δ (2883) cells were grown to mid-exponential phase in EMM2, centrifuged and washed three times in EMM2-N, and cultured in EMM2-N at 25°C. For RNAseq, 2×10^8^ cells were harvested at times 0 and 4 hours, washed with cold DEPC-H_2_0, and snap frozen. RNA extraction was carried out by disrupting the cells with glass beads using RNAeasy Mini kit (Qiagen) and following the manufacturer’s instructions. RNA quality was evaluated using the Bioanalyzer 2100 (Agilent). Library preparation, using the Illumina Ribo Zero and TruSeq Stranded kits, and subsequent NGS sequencing were performed by Macrogen. Sequencing quality was assessed with FastQC (v 0.11.8, Babraham Bioinformatics). If necessary, adaptors were trimmed using Trimmomatic (v 0.38) ^73^. Alignment was performed with HISAT2 v 2.1.0 (CCB in John Hopkins University) ^74^ using *S. pombe* reference genome from Pombase (downloaded on 30/11/2018). Samtools (v 1.9) and deepTools (v 3.3.0) were used to obtain bigWig files to visualize in IGV (v 2.4.16) and JBrowse (v 1.15.4) browsers. Read counts were obtained with featureCounts (Subread package v 1.6.3, Walter+Eliza Hall Bioinformatics) ^75^. DESeq2 (v1.22.2) ^76^ was used for the differential expression analysis. Plots representing upregulated genes in Endosulfine (*igo1*Δ) and Greatwall (*cek1*Δ *ppk18*Δ) mutants shown in Fig. 1a and Supplementary Fig. 1a were generated with karyoploteR (v 1.12.4) ^77^.

For RT-qPCR, total RNA was isolated from 2×10^8^ *S. pombe* cells in exponential phase by disrupting the cells with glass beads in TRIzol^®^ Reagent (Invitrogen) and following the manufacturer’s instructions. The integrity of the RNA was verified through 1% agarose gel electrophoresis, and its quality and quantity were determined using a microspectrophotometer. RNA was treated with RNase-free DNAse I (Invitrogen) at 25°C for 30 minutes, following the manufacturer’s instructions. Each RNA sample (1.2–1.5 μg) was then reverse transcribed with the SuperScript^™^ First-Strand Synthesis System (Invitrogen) using the oligo(dT) primer supplied with the kit or the tRNA^Lys^_UUU_ specific reverse primer in combination with the *act1* gene reverse primer (Supplementary Table 7) at 50°C for 30 minutes in a 20-μl total volume. Quantitative PCR amplification of cDNA (1 μl) was carried out using TB Green Premix Ex Taq^™^ (TaKaRa) and the primer pairs indicated in the Supplementary Table 7, in a 20-μl total volume with the following cycling parameters: 95°C for 45 seconds, 40 cycles of 95°C for 5 seconds and 60°C for 31 seconds, followed by a dissociation step at 95°C for 15 seconds, 60°C for 1 minute and 95°C for 15 seconds. The reactions were run in duplicate or triplicate in an Applied Biosystems 7300 Real-Time PCR System. Negative controls without reverse transcriptase, without RT-primer and without cDNA were included to control for DNA contaminations. Fold changes in the expression levels relative to the wild-type strain grown in EMM2 were calculated according to the mathematic model described by ^78^, with normalization to *act1* expression levels. The experiments were performed at least twice with cDNA from different biological repeats.

### SRFF microscopy

Samples were observed using a Confocal Andor Dragonfly 200 microscope, equipped with a 100x/1.45 Oil Plan Apo objective, an Andor sCMOS Sona 4.2B-11 camera and controlled by Fusion (SRRF-STREAM) software. Image J software was used for general image and movie manipulation. Radial Profile Analysis and the calculations of Pearsońs Correlation Coefficients were performed using ImageJ. More than 100 nuclei were measured for each strain.

### *S. pombe* protein extracts and western blot

TCA extraction was performed as previously described ^79^. For immunoblotting, PVDF membranes were probed with anti-HA (12CA5, Roche), anti-GFP (3H9, Chromotek), anti-GST (RPN1236V, Cytiva) or anti-P-Gad8 (kindly provided by José Cansado, University of Murcia, Spain). Standard procedures were employed for protein transfer, blotting and chemiluminescence detection. Protein detection was performed using the ECL kit (BioRad).

### Chromatin immunoprecipitation (ChIP)

Chromatin isolation and immunoprecipitation was performed as previously described ^80^. *S. pombe* cell cultures were grown in EMM2 or after 4 hours of nitrogen starvation in EMM2-N to OD_600_ of 0.5–0.6 and crosslinked with 1% formaldehyde for 10 min at room temperature. To terminate crosslinking, 2.5M glycine was added to a final concentration of 125 mM for 5 min. Cells were pelleted by centrifugation, washed twice with 10 ml of cold PBS, frozen on dry ice and stored at −80°C. Cell pellets from 50 ml cultures were resuspended in 0.25 ml of Breaking buffer (0.1M Tris-HCl pH 8.0, 20% glycerol, 1 mM PMSF) and lysed in a Fast-prep (2 cycles of 45 s) in the presence of glass beads (50 micron; Sigma) at 4°C. Lysates were centrifuged at 14,000 g for 1 min at 4°C. Pellets were washed with 1 ml of Lysis buffer (50 mM HEPES pH 7.6, 140 mM NaCl, 1 mM EDTA, 1% Triton X-100, 0.1% sodium deoxycholate, 0.1% SDS, 1 mM PMSF). Pellets containing chromatin were resuspended in 0.25 ml of Lysis buffer. Lysates were sonicated for 6 min at 4°C (30 seconds on, 30 seconds off), using a water bath sonicator (Diagenode Bioruptor Plus), transferred to new 1.5-ml Eppendorf tubes, added 0.75 ml of Lysis Buffer and centrifuged at 14,000 g for 30 min at 4°C. 50 μl of supernatant was kept as ‘input’ and the remainder (~950 μl) was subjected to immunoprecipitation with antibodies against K14-acetylated histone H3 (07–353, Upstate Biotechnology) and 20 μl of protein G agarose beads (100.04D, Dynabeads Protein G, Thermo Fisher). After an overnight incubation at 4°C with mixing, beads were washed sequentially with 1 ml of Lysis buffer once, Lysis + 500 mM NaCl twice, Wash buffer (10 mM Tris pH 8.0, 1 mM EDTA, 250 mM LiCl, 0.5% sodium deoxycholate, 0.5% NP-40, 1 mM PMSF) twice, and TE buffer (10 mM Tris pH 7.5, 1 mM EDTA) once. Each wash was for 5 min with mixing at room temperature. Immune complexes were eluted in 100 μl elution buffer (50 mM Tris pH 8.0, 10 mM EDTA, 1% SDS) at 65°C for 20 min. Beads were washed with 150 μl TE + 0.67% SDS, which was combined with the eluate. 150 μl TE + 0.67% SDS was also added to the input samples, and both IP and input samples were incubated at 65°C overnight to reverse protein–DNA crosslinks. DNA was purified by phenol/chloroform extraction. Analysis by qPCR was carried out using a Bio-Rad CFX96 instrument, Takara TB Green premix Ex-Taq, and primers listed in the Key resources table. ChIP signals were calculated as IP/input and normalized to WT 0h with an assigned value of 1.

### Immunoprecipitations and mass-spectrometry analysis

Immunoprecipitation was performed following previously established protocols ^81^. For the immunoprecipitation of Paa1-L-YFP (strain JED62) and Pab1-L-YFP (strain JED56), 1 l of cells was grown to mid-log phase in EMM2, then shifted to EMM2-N for 1 hour and crosslinked with 1% formaldehyde for 10 min at 25 °C. The reaction was quenched by adding glycine to 250 mM and incubating for 5 min on ice. Cells were collected by centrifugation, washed with PBS 1x, frozen in liquid nitrogen and broken with a Freezer/Mill in lysis buffer (25 mM Tris HCl pH 7.5, 150 mM NaCl, 0.5% SDS, 1% NP40, 1 mM PMSF, 1 μg/ml aprotinin, 1 μg/ml leupeptin, 1 μg/ml pepstatin). Cell lysates were then slowly diluted to 0.1% SDS final concentration for immunoprecipitation in lysis buffer without SDS at 4°C for 30 min. Clarified extracts were immunoprecipitated by adding 40 μl of GFP-Trap beads (gta-20, Chromotek) for 1 hour at 4 °C. The beads were washed six times with lysis buffer containing 500 mM NaCl. Finally, the beads were sent to the Proteomics Facility of the Salamanca Cancer Research Center for Mass-spectrometry analysis. Analysis and interpretation of the results were carried out using the String Database.

For the immunoprecipitation of GFP-Pab1 (strain 2895), 16 l of cells were grown to mid-log phase in EMM2 at 25°C. After one day, half of the culture was shifted to EMM2-N for 1 hour. Subsequently, cells were harvested by centrifugation and frozen at −80°C. Cells were lysed using glass beads and a bead beater in 100 ml of NP-40 buffer (6 mM Na_2_HPO_4_, 4 mM NaH_2_PO_4_, 1% NP-40, 150 mM NaCl, 2 mM EDTA, 50 mM NaF and 0.1 mM Na_3_VO_4_) supplemented with complete EDTA-free protease inhibitor cocktail (Roche), 1.3 mM benzamidine (Sigma) and 1 mM PMSF (Sigma). Lysates were cleared by centrifugation, and supernatants were mixed with 60 μl of 50 % slurry GFP-TRAP magnetic agarose beads (GFP-Trap^®^ magnetic agarose, ChromoTek) equilibrated with NP-40 buffer. After 90 minutes of incubation at 4°C, beads were magnetically separated from lysates and washed twice with 5 ml of NP-40 buffer. Samples were washed with 5 ml of low-NP-40 buffer (0.02% NP-40) to reduce total detergent in purified proteins and subsequently resuspended in 1 ml of low-NP-40 buffer. Proteins were eluted twice with 150 μl of elution buffer (200 mM glycine-HCl pH 2.5) and precipitated for 30 minutes on ice using 100 μl of 100% TCA. Samples were then spun down for 30 minutes at 13000 rpm and 4°C, washed with 1 ml of cold acetone containing 0.05 N HCl and 1 ml of cold acetone. Finally, pellets were dried at room temperature and stored at 4°C for mass spectrometry analysis. A small amount of each sample was used to confirmed proper purification of GFP-tagged proteins. For that purpose, the Plus One Silver Staining protein kit (GE Healthcare) was employed following manufacturer instructions. TCA-precipitated proteins were digested with trypsin and analyzed by two-dimensional liquid chromatography tandem MS (2D-LC-MS/MS) as previously described ^82^. MS2 and MS3 spectra were extracted separately from RAW files, and converted to DTA files using Scansifter software ^83^ (v2.1.25). Spectra with less than 20 peaks were excluded and the remaining spectra were searched using the SEQUEST algorithm (Thermo Fisher Scientific, San Jose, CA, USA; version 27, rev. 12). Sequest was set up to search the S. *pombe* protein database (pombe_contams_20151012_rev database, created in October 2015 from pombase.org). Common contaminants were added, and all sequences were reversed to estimate the false discovery rate (FDR), yielding 10390 total entries. Variable modifications (C+57, M+16, [STY]+80 for all spectra and [STY]-18 for MS3), strict trypsin cleavage, <10 missed cleavages, fragment mass tolerance: 0.00 Da (because of rounding in SEQUEST, this results in 0.5 Da tolerance), and parent mass tolerance: 2.5 Da were allowed. Peptide identifications were assembled and filtered in Scaffold (v4.8.4, Proteome Software, Portland, OR) using the following criteria: minimum of 99.0% protein identification probability; minimum of two unique peptides; minimum of 95% peptide identification probability. FDRs were estimated in Scaffold based on the percentage of decoy sequences identified after using the above filtering criteria; the protein level FDR was 0.7% and the peptide level FDR was 0.3%. Proteins containing the same or similar peptides that could not be differentiated based on MS/MS alone were grouped to satisfy the principles of parsimony. Mass spectrometry identified proteins were exported from Scaffold to Excel for further analysis. Further analysis and interpretation of the results was carried out using the String Database (https://string-db.org/*).*

### tRNA purification

tRNA purification assay was performed following established procedures ^8^.

### Quantification of tRNA modifications

The purified tRNAs (500 ng per sample) were subjected to hydrolysis in a 40 μL digestion cocktail containing 10 U benzonase, 4 U calf intestinal alkaline phosphatase, 0.12 U phosphodiesterase I, 0.1 mM deferoxamine, 0.1 mM butylated hydroxytoluene, 4 ng pentosatin, 2.5 mM MgCl_2_ and 5 mM tris buffer (pH 8.0). The digestion mixture was incubated at 37 °C for 6 h. For the verification of HPLC retention times of RNA modifications, synthetic standards were employed. Analytical separation was facilitated by a Thermo Hypersil Gold aQ C18 column (100 × 2.1 mm, 1.9 μm), which was interfaced with an Agilent 1290 HPLC system and an Agilent 6495 triple quadrupole mass spectrometer. The employed LC system operated at 35 °C, maintaining a flow rate of 0.35 mL/min. The gradient starts with 100% solution A (0.1% formic acid in water) for 4 min, followed by a 4–15 min phase involving a transition from 0% to 20% solution B (0.1% formic acid in acetonitrile). The HPLC column was coupled with an Agilent 6495 triple quadrupole mass spectrometer, utilizing an electrospray ionization source in positive ion mode. Operational parameters were set as follows: gas temperature at 120 °C; gas flow rate at 11 L/min; nebulizer pressure at 20 psi; sheath gas temperature at 400 °C; sheath gas flow rate at 12 L/min; capillary voltage at 1500 V; and nozzle voltage maintained at 0 V. The dynamic multiple reaction monitoring mode was used for detection of product ions derived from their respective precursor ions for all the RNA modifications. The collision energy was optimized to ensure maximal detection sensitivity for each modification. To ensure the same sample input, the MS signal intensity for each ribonucleoside was normalized with the UV signal intensity of canonical ribonucleosides. The fold change of the modified ribonucleosides in experiment group was calculated relative to the control group.

### Drug sensitivity assays

For survival on agar plates, *S. pombe* strains were cultured in YES, diluted and the cells were spotted onto plates with minimal medium containing 93.5 mM NH_4_Cl (EMM2) or 20 mM phenylalanine (MMPhe) without or with paromomycin (0.5 mg/ml), puromycin (0.5 mg/ml) or cycloheximide (2.5 μg/ml). The plates were then incubated at the indicated temperatures for 4–8 days.

### Statistical methods

Average, standard deviation, and P values for the two-sided Student’s t test of statistically significant differences were calculated with Microsoft Excel. Data distribution was assumed to be normal, but this was not formally tested.

### Data availability

Further information and requests for reagents may be directed to Sergio Moreno (smo@usal.es) or Javier Encinar del Dedo (jedel_dedo@usal.es).

## Data Availability

The RNAseq data in this study has been deposited in GEO database with the following accession number GSE217398.
